# Synergistic Effect of Zinc Oxide Nanoparticles and *Moringa oleifera* Leaf Extract Alleviates Cadmium Toxicity in *Linum usitatissimum*: Antioxidants and Physiochemical Studies

**DOI:** 10.3389/fpls.2022.900347

**Published:** 2022-08-02

**Authors:** Musarrat Ramzan, Fazila Ayub, Anis Ali Shah, Gul Naz, Adnan Noor Shah, Aqsa Malik, Rehana Sardar, Arkadiusz Telesiński, Hazem M. Kalaji, Eldessoky S. Dessoky, Hamada Abd Elgawad

**Affiliations:** ^1^Department of Botany, The Islamia University of Bahawalpur, Bahawalpur, Pakistan; ^2^Department of Botany, Division of Science and Technology, University of Education, Lahore, Pakistan; ^3^Faculty of Science, Institute of Physics, The Islamia University of Bahawalpur, Bahawalpur, Pakistan; ^4^Department of Agricultural Engineering, Khwaja Fareed University of Engineering and Information Technology, Punjab, Pakistan; ^5^Department of Botany, University of Narowal, Narowal, Pakistan; ^6^Institute of Botany, University of the Punjab, Lahore, Pakistan; ^7^Zachodniopomorski Uniwersytet Technologiczny w Szczecinie, West Pomeranian University of Technology, Szczecin, Poland; ^8^Department of Plant Physiology, Institute of Biology, Warsaw University of Life Science, Warsaw, Poland; ^9^Institute of Technology and Life Sciences - National Research Institute, Raszyn, Poland; ^10^Department of Biology, College of Science, Taif University, Taif, Saudi Arabia; ^11^Botany and Microbiology Department, Faculty of Science, Beni-Suef University, Beni-Suef, Egypt

**Keywords:** ZnO-NPs, cadmium, linseed, antioxidant, plants

## Abstract

Among heavy metals, cadmium (Cd) is one of the toxic metals, which significantly reduce the growth of plants even at a low concentration. Cd interacts with various plant mechanisms at the physiological and antioxidant levels, resulting in decreased plant growth. This research was conducted to exploit the potential of synergistic application of zinc oxide nanoparticles (ZnO NPs) and *Moringa oleifera* leaf extract in mitigation of Cd stress in linseed (*Linum usitatissimum* L.) plants. The main aim of this study was to exploit the role of *M. oleifera* leaf extract and ZnO NPs on Cd-exposed linseed plants. Cd concentrations in the root and shoot of linseed plants decreased after administration of MZnO NPs. Growth parameters of plants, antioxidant system, and physiochemical parameters decreased as the external Cd level increased. The administration of MZnO NPs to the Cd-stressed linseed plant resulted in a significant increase in growth and antioxidant enzymes. Furthermore, the antioxidative enzymes superoxide dismutase (SOD), peroxidase (POD), catalase (CAT), and ascorbate peroxidase (APX) exhibited a considerable increase in the activity when MZnO NPs were applied to Cd-stressed seedlings. The introduction of MZnO NPs lowered the levels of malondialdehyde (MDA) and hydrogen peroxide (H_2_O_2_) in the linseed plant grown in Cd-toxic conditions. The NPs decreased electrolyte leakage (EL) in Cd-stressed linseed leaves and roots. It was concluded that synergistic application of ZnO NPs and *M. oleifera* leaf extract alleviated Cd stress in linseed plants through enhanced activity of antioxidant enzymes. It is proposed that role of MZnO NPs may be evaluated for mitigation of numerous abiotic stresses.

## Introduction

Heavy metal contamination in the ecosystem has been studied extensively and has attained a lot of attention due to the substantial health risks that these metals pose to terrestrial and marine ecosystems (Ali et al., 2014; Mwamba et al., 2016). Toxic effects of heavy metals to humans include nervous disorder, vascular damage, birth defects, and kidney dysfunction. Lead (Pb), cadmium (Cd), and chromium (Cr) are the most dangerous nonessential metals (Sebastiani et al., 2004; Ali et al., 2019). Cd is a “priority contaminant” among these, causing potential hazards not just to public health but also to a broad ecosystem (Campbell, 2006; Ali et al., 2015). It is another extensively distributed heavy metal whose transport into the atmosphere and plant-soil natural environment is mostly caused by industrial operations, mining operations, solid wastes, municipal wastewater, and use as fertilizer in farm fields (Rao et al., 2010).

Cadmium poisoning raises the oxidative stress while deteriorating chloroplast structures, reducing the rate of photosynthesis in damaged plants. Higher reactive oxygen species (ROS) levels induce nucleotide breakdown, protein oxidation, and lipid peroxidation (Zaheer et al., 2018; Mwamba et al., 2020). Cd is promptly absorbed by the roots and can be transported into the xylem for transfer to the leaves. The quantity of Cd deposited in roots or translocated to leaves varies greatly between species (Ali et al., 2013a,b). Most plants are susceptible to low Cd concentrations, which restrict root and shoot growth due to changes in the photosynthetic rate, macronutrient absorption, and micronutrient distribution (Shi et al., 2005).

Nanotechnology is a contemporary scientific discipline concerned with nanoscale materials. It is an emerging field that has been rapidly evolving and works in everyday life as a result of the significant impact that its vast applications have in all aspects of human life (Khan et al., 2020; Zhang, 2020). Nanomaterials, which are molecular and atomic aggregates with a diameter of lower than 100 nm, are the focus of nanotechnology. These are modified analogs of essential elements created by changing their molecular and atomic characteristics (Kato, 2011). Metal-based nanoparticles (NPs) are the most researched nanomaterials among the many nanomaterials (Lin et al., 2009). Nanomaterials increase soil fertility by chelating various ions and salts available in the soil, regulating soil pH, and interacting with soil microbes, indirectly assisting better growth of plants (Fraceto et al., 2016). Furthermore, in the agricultural field, NPs are used as a quick diagnostic tool to check different challenges of environment such as drought and salinity and to determine the level of soil nutrients and heavy metals (Alghuthaymi et al., 2015).

Zinc is a micronutrient that is required for humans, wildlife, and crop production. The deficiency of zinc can result in stunted growth in plants and loss of yield (Merchant, 2010). Zinc oxide (ZnO) NPs are widely employed in medical fields and other applications due to their antibacterial qualities, with roughly 528 tons produced globally each year (Gümüş et al., 2014). In current years, application of micro and macronutrients in the form of NPs has been regarded as a viable method for enhancing the crop growth and yield in most crops (Rizwan et al., 2017a,b, 2019a,b). These NP supplements may assist to prevent nutrient loss and increase crop output in a sustainable way (Dimkpa et al., 2017). Several scientists investigated the effect of ZnO NPs on various crop plants, and their findings indicate that ZnO NPs have a good effect on plant growth. It has also been found that the harmful effects of ZnO NPs on agricultural crops are substantially lower than that of Zn^2+^ or bulk particles (i.e., ZnO) (Kouhi et al., 2015). When compared with the ionic form (i.e., Zn^2+^) of the equivalent metal and micro (macro) particles, foliar-applied ZnO NPs concentrations can have both favorable and harmful effects on the development of plants (Mousavi Kouhi et al., 2014).

*Moringa oleifera* belongs to the monogeneric Moringaceae family and is also known as sohanjana, drumstick, and horseradish tree (Marrufo et al., 2013). *Moringa oleifera* a tiny, rapidly growing ornamental tree found throughout Africa and Asia's tropical climates (Sreelatha et al., 2011). Moringa leaves has been observed to be a rich source of carotene, protein, vitamin C, calcium, and potassium, as well as a good source of natural antioxidants (Moyo et al., 2012). In recent years, extracts of *M. oleifera's* leaves, seeds, and roots have been extensively researched for a variety of possible applications, such as wound healing, antihepatotoxicity, antifertility, hypotensive, and analgesic action (Karadi et al., 2006).

Linseed, generally known as common flax, is a fibrous crop and dicotyledonous plant in the Linaceae family with economic values (Feller et al., 2019). It is a white or a blue-flowered plant that has traditionally been used for fiber and food in cooler parts of the world. It is an annual herbaceous plant. Linseed has been farmed for 5,000 years in China and India and 10,000 years ago in Egypt and Samaria (Akhtar et al., 2013; Wiszniewska et al., 2016). Flaxseeds provide numerous nutritional benefits and a higher proportion of short-chain omega-3 fatty acids. To determine whether *M. oleifera*-stabilized ZnO NPs can minimize Cd toxicity in the linseed plant. The study was conducted to evaluate the potential for synergistic application of ZnO NPs and *M. oleifera* in alleviation of Cd stress in linseed.

## Materials and Methods

### Preparation of *Moringa oleifera* Extract

Cholistan Institute of Desert Studies (CIDS)/The Islamia University of Bahawalpur (IUB) provided the powder form of washed and dried *M. oleifera* leaves. To prepare the leaves extract, a 2.8 g of *M. oleifera* powder was added to 100 ml of deionized water and then heated at 90°C for ~1 h using a hotplate under continuous stirring condition. After being cooled at room temperature, the extract was filtered using the Whatman No. 1 filter paper to remove the residues. The filtrate was stored in a glass bottle at 4°C for further usage. The as-prepared *M. oleifera* green extract contains various biological elements such as alkaloids, flavonoids, phenols, gallic acid, and amino acids. These elements play a vital role in the preparation and stability of *M. oleifera*-reduced ZnO NPs (Patel et al., 2012).

### Synthesis of *Moringa oleifera*-Stabilized Zinc Oxide Nanoparticles

**Figure 1:**
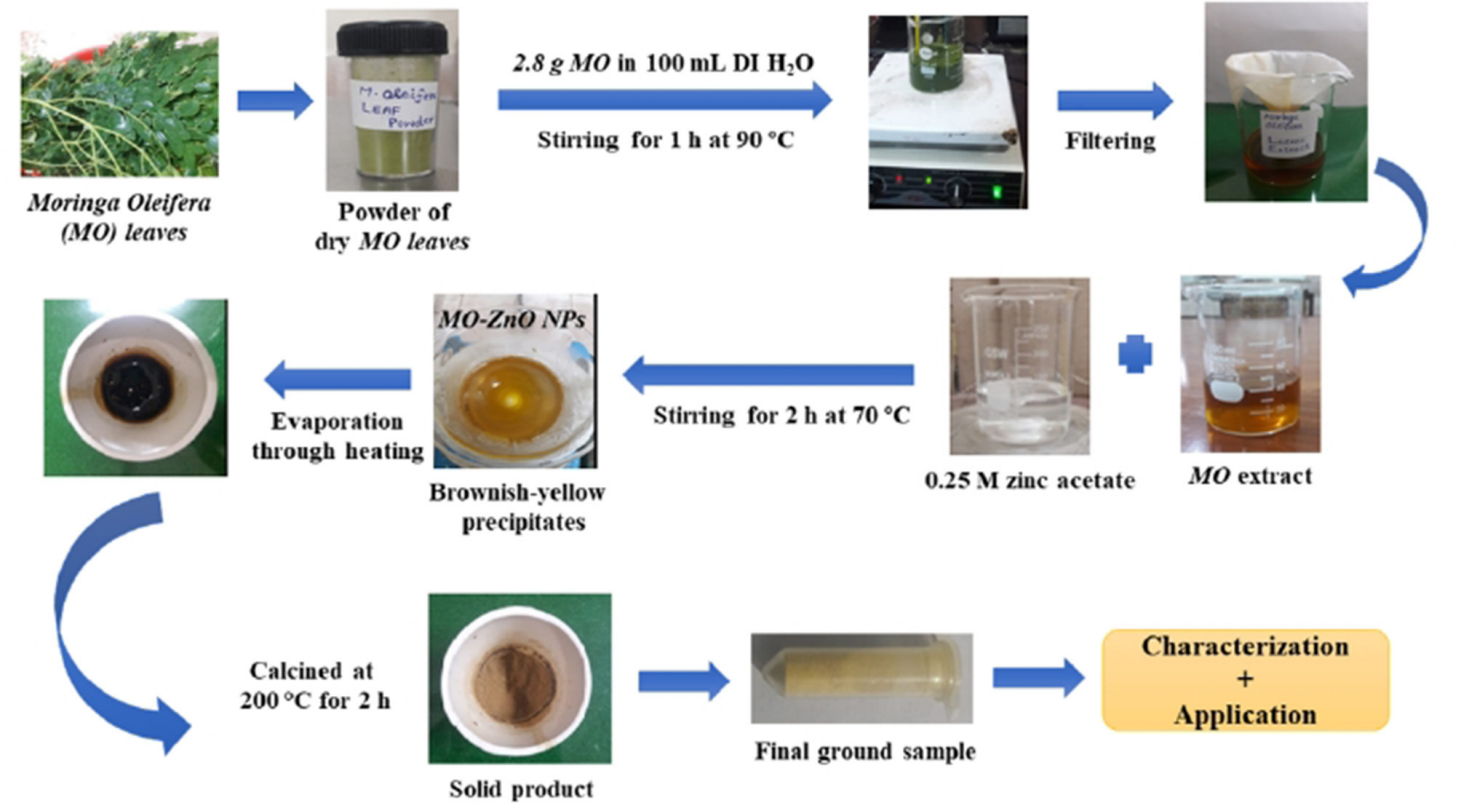
Schematic of the synthesis of *Moringa Oleifera* (*MO*) leaves-stabilized ZnO NPs.

For *M. oleifera* extract-mediated synthesis of ZnO NPs, deionized H_2_O was used as a solvent throughout the procedure. On a laboratory hot plate, 4.5 g of zinc acetate dehydrate/Zn(CH_3_CO_2_)_2_.2H_2_O (Sigma Aldrich, 99.99% purity) was dissolved in 100 ml of deionized water with continuous stirring to make a 0.25 M Zn stock solution. The obtained *M. oleifera* leaves extract was then added to the zinc acetate stock solution, which was continuously stirred with a magnetic stirrer while heating at 70°C for 2 h. The resultant solution was evaporated on a hot plate, and the leftover solid sample or paste-like substance was placed in a ceramic crucible cup and calcined for 2 h at 200°C in a muffle furnace to eliminate impurities. Following that, changes in the color of the sample indicate the formation of *MO*-ZnO NPs (Mahendiran et al., 2017). A yellowish-brown-colored solid product was obtained, which was ground in a sterilized mortar/pestle system to produce powdered *M. oleifera*-ZnO NPs. Several early experiment trails were performed to get this optimal concentration of zinc acetate and plant extract (Figure 1).

### Characterization

To confirm the morphology and stability of *M. oleifera*-stabilized ZnO NPs, the absorption spectrum of the as-prepared sample was obtained using an Epoch Microplate Spectrophotometer in 300–700 nm. The X-ray diffraction (XRD) for this sample was performed through the Bruker-D8 Advance X-ray Diffractometer with Cu *K*α radiation (λ = 1.54 Å), operated at 35 mA and 40 keV. The machine scanned the sample at room temperature from 20° to 85° for its structural analysis. To examine the surface morphology of *M. oleifera*-stabilized ZnO NPs, a JEOL SEM (made in Japan) was used. An X-ray analysis option, energy-dispersive X-ray (EDX) equipped with the scanning electron microscope (SEM), was utilized to determine the elemental/quantitative compositional information of the as-synthesized sample.

### Experiment Design

Linseed (*Linum usitatissimum)* was selected for the experiment. Viable seeds were selected and used for greenhouse experiment. Cd was purchased from the Sigma International Company. Different concentrations of Cd were prepared for further experimentation. This experiment was carried out at the Department of Botany and Wire house of Horticulture Department, the Islamia University of Bahawalpur. The soil was collected from the local Nursery of Bahawalpur for the greenhouse experiment. The Bhal soil was washed *via* a 2 mm steel sieve after being acquired from a local Bahawalpur nursery. Cd in the form of Cd chloride (CdCl_2_) was introduced into the test soil. For Cd contaminations, soil was spiked with 100 mg/kg Cd, whereas noncontaminated soil was used as control. To achieve a balance between the soil's solid phase and liquid phase, contaminated soils were stored in the shade for 1 month prior to seed sowing. The experiment was carried out in a completely randomized design (CRD). Each treatment composed of 3 replicates. Each pot contained 3 plants. Greenhouse conditions during the experiment were 30°C average temperature, 16 h day/8 h night photoperiod, 80% relative humidity, and 200 μmol m^−2^ s^−1^ light intensity.

### Application of *Moringa oleifera* Leaf Extract and Zinc Oxide Nanoparticles on Linseed

Three concentrations of NPs, namely, 100 mg/L, 500 mg/L, and 1 g/L, were dissolved in 1 L (1,000 ml) of distilled water and mixed well and sprayed on 1 month seedling of linseed. *Moringa oleifera*-grinded leaves extract was also sprayed on linseed. In total, six to eight linseed seeds were sown at equal distances in 1.5 kg of soil. Plastic containers were used to propagate the seeds. After 1 week, the germinating plants were thinned. The plants were irrigated regularly to maintain a moisture content of 40% throughout all containers. All plants were studied until constant heights, and those plants with constant heights were harvested after 3 months. Shoots were taken from the roots, and the roots were cleaned with distilled water to eliminate particles of soil. The plant tissue and soil were oven dried at a temperature of 400°C. The fresh and dry plant root and shoot weights were measured and reported as root and shoot biomass.

### Estimation of Plant Growth Attributes

Using the measuring scale, morphological parameters were determined. Shoot and root fresh weight (FW) and dry weight were computed in grams, while root and shoot length was measured in cm. The dry mass of shoots and roots was recorded from 9-day-old seedlings after keeping them in a scientific oven at 70°C for 75 h.

### Estimation of Chlorophyll and Carotenoids Contents

The concentration of photosynthetic pigment (i.e., chlorophyll a, b, and total chlorophyll) was quantified using the Lichtenthaler method (1987). The pigment extract was quantified against a blank of acetone at wavelengths of 646 nm and 663 nm for chlorophyll assays and 470 nm for carotenoids, and all pigments were expressed as mg g^−1^ FW leaf.

### Estimation of Leaf Relative Water Content

The completely expanded leaves specimens were immersed in 100 ml of sterilized distilled water for 24 h in a dark area at 10°C. These turgid leaf samples were quickly wrapped in blotting paper for 2 min before being weighed. Samples of leaves were oven dried at 75°C for 2 days, and LRWC was calculated using the Smart and Bingham (1974) method.

### Estimation of Antioxidant Enzyme

To evaluate SOD activity, a piece of 0.2 g fresh leaves was put in an ice bucket and crushed with 1% polyvinylpyrrolidone (PVP) in the presence of phosphate buffer at pH 7.0. This mixture was centrifuged at 4°C for 25 min at 15,000 *g*. The supernatant superoxide dismutase (SOD) characteristic was examined by Giannopolitis and Ries (1977) by measuring the enzyme that generated 50% suppression of nitroblue tetrazolium chloride in the presence of riboflavin at 560 nm.

Fresh leaf specimens (1 g) were well agitated with a 4 ml extractor buffer comprising 1 mM phenylmethylsulfonyl fluoride (PMSF), 1% PVP, 1 mM ethylenediaminetetraacetic acid (EDTA), and 50 mM phosphate buffer at pH 7. Upadhyaya et al. (1985) used the resultant supernatant to test peroxidase (POD) operation after centrifugation at 15,000 rpm for 30 min at 4°C. Guaiacol oxidation enhanced the absorbance values measured at 420 nm.

For catalase (CAT) analysis, 0.5 g of fine powder from dried oven leaves was mixed in an ice-cold pestle and mortar with 1 mM EDTA, 1 mM ascorbic acid, 2% PVP (w/v), and 0.05% Triton X-100 (w/v) in 50 mM potassium phosphate buffer (2 ml) at pH 7. According to Aebi (1983), after centrifuging the solution at 1,000 rpm for 20 min at 4°C, the resultant supernatant was utilized to assess the CAT enzyme activity. The lowered absorbance rate of H_2_O_2_ was spectrophotometrically measured at 240 nm to determine the CAT behavior. As a result, for 2 min, 10.5 mM H_2_O_2_ and 50 mM potassium phosphate buffer at pH 7 were added to the 25°C enzyme extract.

For APX estimation, 0.2 ml of the solution was mixed with 0.1 mM EDTA, 0.25 mM ascorbic acid, and 25 mM phosphate buffer at pH 7. The first reading for ascorbate oxidation was obtained after the addition of 1 mM H_2_O_2_, and the second reading was obtained at 290 nm after 1 min interval using a spectrophotometer. The extinct value distinguished the differences in ascorbate oxidation values.

### Estimation of Electrolyte Leakage in Leaves

The completely developed leaves (2) were sliced into 0.5 cm pieces and immersed in 7 ml sterilized water in a glass container containing leaf segments before being placed in a rotatory shaker at 120°C for 30 min. The reading for primary leaf conductivity (EC-i) was derived by autoclaving the sample for 30 min at 120°C and then cooling at room temperature (maximum value) or final conductivity (EC-f) by formula (Li et al., 2018),


(1) $$\begin{array}{l} \text{E}\text{L}=\left(\text{E}\text{C}-\text{i}\right)/\left(\text{E}\text{C}-\text{f}\right)\times 100 \end{array}$$

### Evaluation of Hydrogen Peroxide

The fresh leaf extract (0.25 g) of treated plants was homogenized with 5% trichloroacetic acid (TCA, 3 ml) in the presence of activated charcoal (0.1 g) at 0°C, followed by centrifugation at 12,000 rpm for 15 min. The supernatant was then mixed with 1 M potassium iodide (0.75 ml) and 10 mM potassium phosphate buffer (0.5 ml) at pH 7.0. The spectrophotometric values of the solution were measured at 390 nm and correlated with known H_2_O_2_ concentration (Velikova et al., 2000).

### Measurement of Malondialdehyde Content

The malondialdehyde content was determined using the thiobarbituric acid reaction reported by Rao and Sresty (2000). A fresh 0.5 g specimen of prewashed leaves was vortexed with 10 ml of trichloroacetic acid (0.1% w/v) and centrifuged for 15 min at 4°C in an ice bath. The supernatant (2 ml) and 2 ml thiobarbituric acid (0.67% w/v) solution were produced and stored at 100°C. Following 0.5 h, the supernatant was transferred to the ice bath. The precooled solution (4°C) was centrifuged at 10,000 *g* for 30 s, followed by absorbance measurement of the supernatant at 532 nm. Nonspecific absorption was removed from the 600 nm reading. The MDA concentration was determined using the MDA molar extinction coefficient.

### Estimation of Proline Content

Shaking for 30 min at 150 rpm, the freeze-dried powder (0.1 g) was extracted in 3 ml of 3% sulfosalicyclic acid dihydrate. The supernatant was centrifuged at 2,400 g for 1 min and then passed through a 0.2 m syringe filter with a 1 ml syringe and stored. The supernatant was then diluted with 3% sulfosalicyclic acid dehydrate, and the proline content was determined with a spectrophotometer using the method published by Bates et al. (1973).

### Estimation of Total Soluble Protein Content

The protein content was assessed using a standard curve established by distinct concentrations of bovine serum albumin (BSA). Furthermore, 1 ml of sample plant leaf extract was placed in a test tube. Phosphate buffer was mounted at 1 ml with pH 7.0 in log. The test tubes containing the reagents were held at room temperature for 1 min. A 0.5 ml of Folin-phenol reagent was combined and incubated for 30 min. The optical range was read by a spectrometer at 620 nm.

### Estimation of Total Soluble Sugar

The Yemm and Willis (1954) method was used to determine the maximum soluble sugars. In 25 ml test tubes, 0.1 ml of plant extract was collected. Anthrone reagent (6 ml) was used in each tube, which was then immersed in a boiling bath for 10 min to heat up. The test tubes were solidified at room temperature for 10 min before being incubated for 20 min. The spectrophotometric value was determined at 25nm.

### Statistical Analysis

The data were entered into the computer software in this investigation. The arithmetic mean and standard error were computed. To compare different treatments, a one-way ANOVA test was used. To make multiple comparisons between averages of different treatments, the one-way ANOVA test was used followed by a *post-hoc* test using the Duncan's test.

## Results

**Figure 2:**
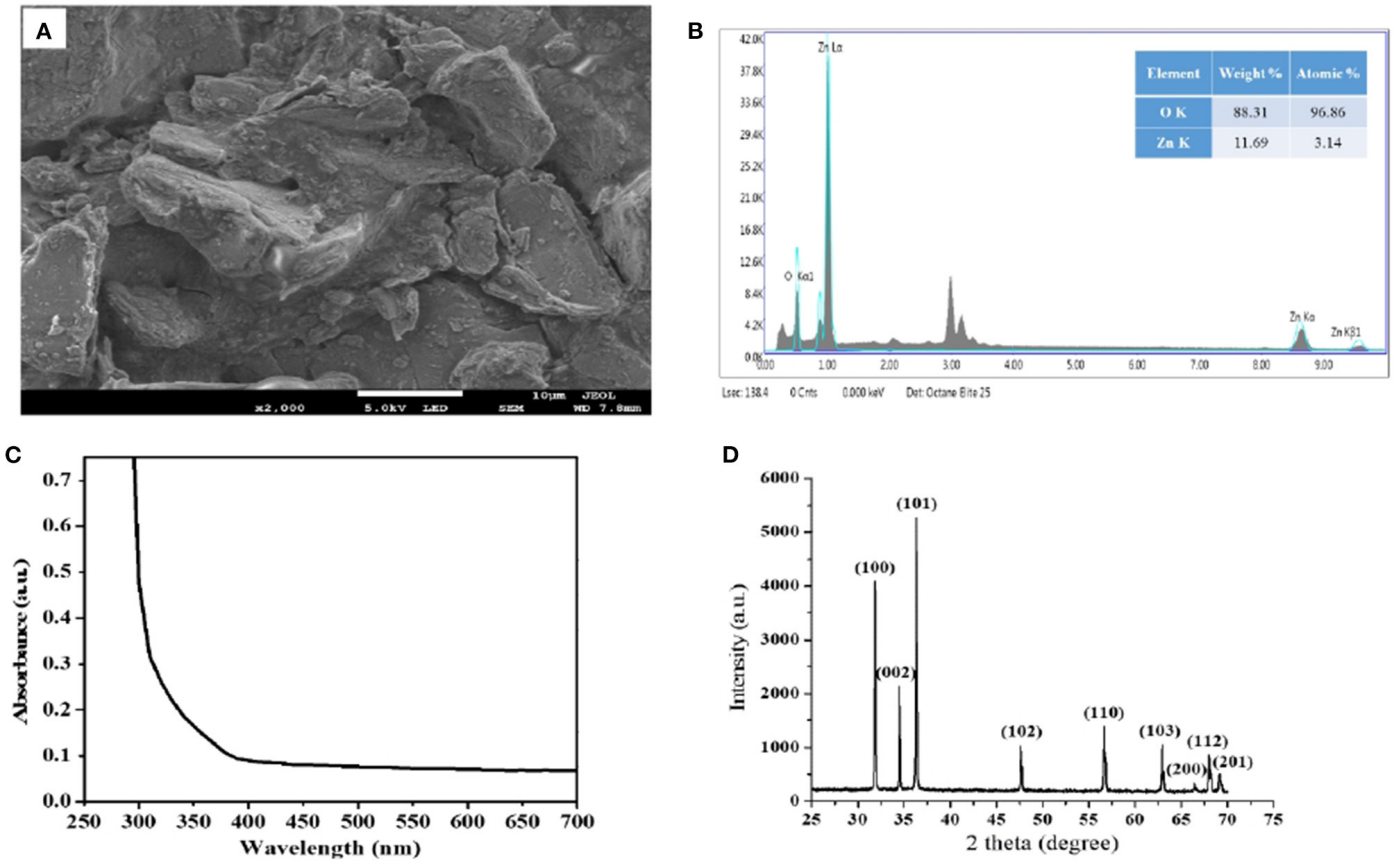
**(A)** SEM image of *MO*-stabilized ZnO NPs exhibiting nano-granules accumulated on the bulk, **(B)** EDX map clearly showing traces of Zn and oxygen elements, **(C)** corresponding UV-Vis absorption spectrum and **(D)** XRD pattern of *MO*-stabilized ZnO NPs.

To estimate the dimensions and shape of *M. oleifera*-stabilized ZnO NPs, the SEM analysis is performed and the micrograph is shown in Figure 2A. It is depicted that the sample is incorporating small granules of sizes in the nano-regime. It seems that the NPs sample could not be fine-ground before the SEM analysis. The size estimate of these nano-granules falls below 700 nm.

Figure 2B shows corresponding EDX spectroscopic analysis of *M. oleifera*-stabilized ZnO NPs, noticeably indicating the peaks for Zn and oxygen elements. The EDX spectrum of our sample obtained from the SEM-EDX analysis shows that the sample prepared by using *M. oleifera* leaves extract has pure ZnO phases, and the reaction product comprises of high-purity ZnO NPs. The approximate atomic ratio of Zn to oxygen is found to be 3.14:96.86, as shown in Figure 2B.

Figure 2C shows the UV-Vis absorption spectrum of *M. oleifera*-stabilized ZnO NPs. Usually, pristine ZnO does not show any absorption peak in the visible region, but an equitable absorption band can be observed in the UV region, which may be attributed to the band edge absorption of hexagonal wurtzite crystalline structure of ZnO material (Alenezi et al., 2013). Figure 2D shows the XRD pattern of *M. oleifera*-stabilized ZnO NPs, where the diffraction peaks are obvious to locate at 2θ values of 31.852, 34.511, 36.331, 47.62, 56.66, 62.917, 66.435, 67.99, and 69.175° corresponding to (100), (002), (101), (102), (110), (103), (200), (112), and (201) crystalline planes, respectively. This is in agreement with the previously reported literature being indexed to the hexagonal wurtzite type of ZnO material (JCPDS 36-1451) (Wang et al., 2017). The perception of the XRD pattern reflects the crystalline quality of *M. oleifera*-stabilized ZnO NPs. No other diffraction peaks have been observed in this pattern, indicating the absence of any secondary phases of our sample.

### Estimation of Plant Growth Attributes

As demonstrated in Table 1, fresh shoot weight (FSW) did not decrease much in stressed plants treated for Cd stress. The application of 2.8 g/100 ml *Moringa* leaf extract (MLE) on the plant decreased the FSW compared with control and Cd-stressed plants, whereas under Cd stress, *M. oleifera* leaf extract increased the FSW. The application of NP1 (100 mg/L) alone significantly increased the FSW, whereas other levels of MZnO NPs, i.e., NP2 (500 mg/L) and NP3 (1 g/L), did not significantly increase the FSW in nontreated plants. Dry shoot weight (DSW) did not decrease much in stressed plants treated to Cd stress as shown in Table 1. The application of NP1 (100 mg/L) alone significantly increased the DSW in nonstressed plants, whereas other levels of MZnO NPs NP2 (500 mg/L) and NP3 (1 g/L) moderately increase the DSW. Fresh root weight declined in Cd-stressed plants as compared with control. The application of 2.8 g/100 ml *Moringa oleifera* leaves (M.L) powder decreased the FRW and DRW compared with control and Cd-stressed plants. The application of MZnO NPs alone significantly increased the fresh root weight, while an increased level of MZnO NPs (1 g/L) decreased the fresh root weight. The application of 2.8 g/100 ml *M. oleifera* leaf extract alone on the nontreated plant decreased the shoot length compared with the control and Cd-stressed plants. In contrast, under Cd stress, MLE increases the shoot length. The root length decreased at zero concentration of MZnO NPs (0 mg/L). Supplementation of *M. oleifera* leaf extract under Cd stress increased the root length. The application of NPs NP1 (100 mg/L) alone significantly enhanced root length followed by NP2 (500 mg/L).

**Table 1:** Effects of MZnO nanoparticles (NPs) on shoot length, root length, fresh shoot weight, fresh root weight, dry shoot weight, and dry root weight under Cd stress in linseed.

**Treatments**		**Growth traits**				
	**Shoot length (cm)**	**Root length (cm)**	**FSW (g plant^**−1**^)**	**FRW (g plant^**−1**^)**	**DSW (g plant^**−1**^)**	**DRW (g plant^**−1**^)**
C	32 ±0.13bc	2 ± 0.3d	0.15 ± 0.09d	0.018 ± 0.13d	0.04 ± 0.08e	0.02 ± 0.06b
Cd	28.3 ±0.2de	1.4 ± 0.02e	0.173 ± 0.04cd	0.013 ± 0.8e	0.042 ± 0.09f	0.003 ± 0.03de
M.L	24.1 ±0.09f	2 ± 0.19dc	0.124 ± 0.11e	0.012 ± 0.20f	0.02 ± 0.11c	0.002 ± 0.31f
Cd + M.L	32.7 ±0.11bc	2.2 ± 0.13d	0.152 ± 0.14*d*	0.014 ± 0.15e	0.04 ± 0.14a	0.003 ± 0.09de
NP1	35.2 ±0.07b	4.1 ± 0.4a	0.48 ± 0.01a	0.031 ± 0.20c	0.112 ± 0.10de	0.008 ± 0.16c
NP2	41.5 ±0.12a	3.4 ± 0.14c	0.24 ± 0.05b	0.064 ± 0.15a	0.093 ± 0.15de	0.008 ± 0.21c
NP3	30.2 ±02a	2 ± 0.21dc	0.181 ± 0.25c	0.012 ± 0.11*f*	0.052 ± 0.23d	0.013 ± 0.15a
Cd + NP1	35 ±0.12b	3.6 ± 0.09b	0.13 ± 0.15f	0.016 ± 0.03ef	0.054 ± 0.17b	0.003 ± 0.04de
Cd + NP2	25 ±0.11e	1.8 ± 0.11f	0.05 ± 0.41g	0.036 ± 0.08b	0.034 ± 0.20e	0.004 ± 0.06d
Cd + NP3	30 ±0.03d	4.2 ± 0.01a	0.091 ± 0.07g	0.009 ± 0.21g	0.033 ± 0.04e	0.003 ± 0.06de

*Different letters indicate significant difference between the treatments. Data are means ± SE (n = 5). Nonidentical letters specify significant difference at P ≤ 0.05. C, control; Cd, 100 mg/kg Cd; M.L, Moringa oleifera leaves; NP1, 100 mg/L MZnO NPs; NP2, 500 mg/L MZnO NPs; NP3, 1 g/L MZnO NPs*.

### Evaluation of Chlorophyll a, b and Carotenoids in Linseed Shoots Under Cadmium Stress

In this study, MZnO NPs and *M. oleifera* leaf extract significantly impacted the Chl *a* parameter compared with control as shown in Table 2. The lower concentration of Chl *a* and *b* was observed in Cd-alone-treated seedlings. The application of NP1 (100 mg/L) to Cd-stressed plants enhanced the Chl *a* in contrast to those plants treated with Cd alone. The treatments (500 mg/L and 1 g/L) of MZnO NPs also enhanced Chl *a* over those in Cd-alone-treated plants. In this study, MZnO NPs and *M. oleifera* leaf extract significantly impacted the Chl *b* parameter compared with control. The treatment (500 mg/L and 1 g/L) of MZnO NPs also enhanced total Chl over those in Cd-alone-treated plants. The results of this study depicted that carotenoid amount significantly increased in control seedlings over Cd-treated seedlings. Nonsignificant carotenoids amount was found in the 2.8 g/100 ml *M. oleifera* leaf extract supplemented seedlings over Cd-stressed plants. However, among NPs treatments, it was found that NP2 (500 mg/L) enhanced carotenoids in nonstressed plants.

**Table 2:** Effects of MZnO nanoparticles (NPs) on Chl *a*, Chl *b*, total chl, carotenoids, and leaf relative water content under Cd stress in linseed.

**Treatments**	**Chl *a* (mg/g FW)**	**Chl *b* (mg/g FW)**	**Total Chlorophyll**	**Carotenoids**	**LRWC**
C	0.312 ± 0.021c	0.185 ± 0.04b	0.497 ± 0.06c	0.415 ± 0.3a	30 ± 0.06d
Cd	0.256 ± 0.014d	0.134 ± 0.02f	0.391 ± 0.03e	0.328 ± 0.031b	20 ± 0.09e
M.L	1.339 ± 0.09a	0.214 ± 0.07g	0.553 ± 0.01a	0.252 ± 0.14de	13.1 ± 0.21g
Cd + M.L	1.315 ± 0.09b	0.198 ± 0.04a	0.513 ± 0.40b	0.215 ± 0.31a	5 ± 0.05a
NP1	0.312 ± 0.02bc	0.178 ± 0.08cd	0.49 ± 0.07c	0.255 ± 0.19de	13.7 ± 0.12g
NP2	0.296 ± 0.03d	0.165 ± 0.01c	0.461 ± 0.04cd	0.274 ± 0.17d	52.9 ± 0.07b
NP3	0.312 ± 0.01c	0.174 ± 0.03cd	0.486 ± 0.11cd	0.231 ± 0.22e	11.5 ± 0.15h
Cd + NP1	0.312 ± 0.06c	0.185 ± 0.02b	0.497 ± 0.07c	0.254 ± 0.41e	15.8 ± 0.30f
Cd + NP2	0.274 ± 0.04e	0.153 ± 0.04d	0.427 ± 0.02d	0.232 ± 0.32f	56 ± 0.03a
Cd + NP3	0.235 ± 0.019f	0.142 ± 0.08e	0.377 ± 0.08f	0.315 ± 0.02c	47.8 ± 0.06c

*Different letters indicate significant difference between the treatments. Data are means ± SE (n = 5). Nonidentical letters specify significant difference at P ≤ 0.05. C, control; Cd, 100 mg/kg Cd; M.L, Moringa oleifera leaves; NP1, 100 mg/L MZnO NPs; NP2, 500 mg/L MZnO NPs; NP3, 1 g/L MZnO NPs*.

### Evaluation of Leaf Relative Water Content in Linseed Under Cadmium Stress

Leaf water content decreased in Cd-stressed plants compared with control as shown in Table 2. However, levels of leaf water content decreased when plants were treated with *M. oleifera* leaf extract (2.8 g/100 ml). The application of MZnO NPs (500 mg/L) enhanced leaf water content in plants. Under Cd stress, MZnO NPs increased the leaf relative water content.

### Evaluation of Soluble Sugar in Linseed Root and Shoots Under Cadmium Stress

**Figure 3:**
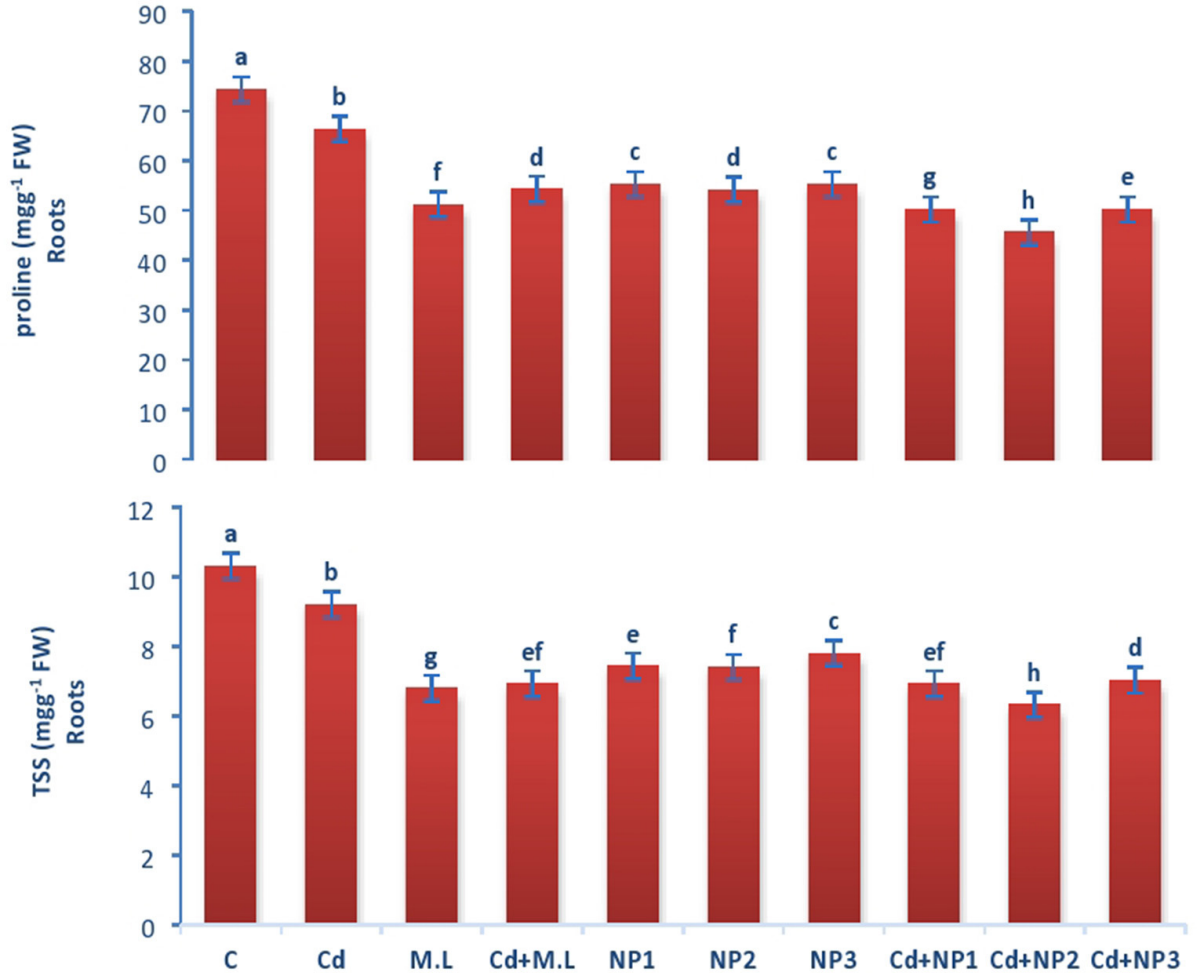
Effects of MZnO–NPs on protein, proline, and TSS under Cd stress in Linseed roots. Different letters indicate significant difference between the treatments. Data are means ± SE (*n* = 5). Non-identical letters specify significant difference at *P* ≤ 0.05. C, Control; Cd, 100 mg kg^−1^ Cd; M. L, *Moringa oleifera* leaves; NP1, 100 mg L^−1^ MZnO-NPs; NP2, 500 mg L^−1^ MZnO-NPs; NP3, 1 g L^−1^ MZnO-NPs.

The levels of total soluble sugar in Cd-treated plants and without stressed plants were found to be declined when they were treated with 2.8 g/100 ml *M. oleifera* leaf extract only in contrast to those plants subjected to Cd alone (Figure 3). The supplementation of alone MZnO NPs does not show any significant difference in total soluble sugar amount of root. The amount of total suspended solid (TSS) in shoots enhanced in nonstressed plants in contrast to Cd-stressed plants as shown in Figure 2. The levels of total soluble sugar in Cd-treated plants and without stressed plants were found to be declined when they were treated with 2.8 g/100 ml MLE in contrast to those plants treated with Cd only. The amount of total soluble sugar increased under Cd stress compared to that in the M.L-supplemented plants.

### Evaluation of Total Soluble Protein in Linseed Root and Shoots Under Cadmium Stress

The levels of total soluble protein in roots increased when plants were supplemented with 2.8 g/100 ml *M. oleifera* leaf extract. The amount of protein is different among all the MZnO NPs levels. However, enhancement in the level of protein was observed in NP2 (500 mg/L) MZnO NPs, under Cd stresses compared with that in the plants supplemented with MZnO NPs alone. The amount of total soluble protein in shoots enhanced in control in relation to Cd-stressed plants as shown in Figure 3. However, the levels of total soluble protein increased when plants were supplemented with 2.8 g/100 ml *M. oleifera* leaf extract. The amount of total soluble protein is not significantly different among all the MZnO NPs levels. However, enhancement in the level of protein was observed in NP2 (500 mg/L) MZnO NPs under Cd stresses compared with that in the plants supplemented with alone MZnO NPs.

### Evaluation of Proline Content in Linseed Shoots Under Cadmium Stress

**Figure 4:**
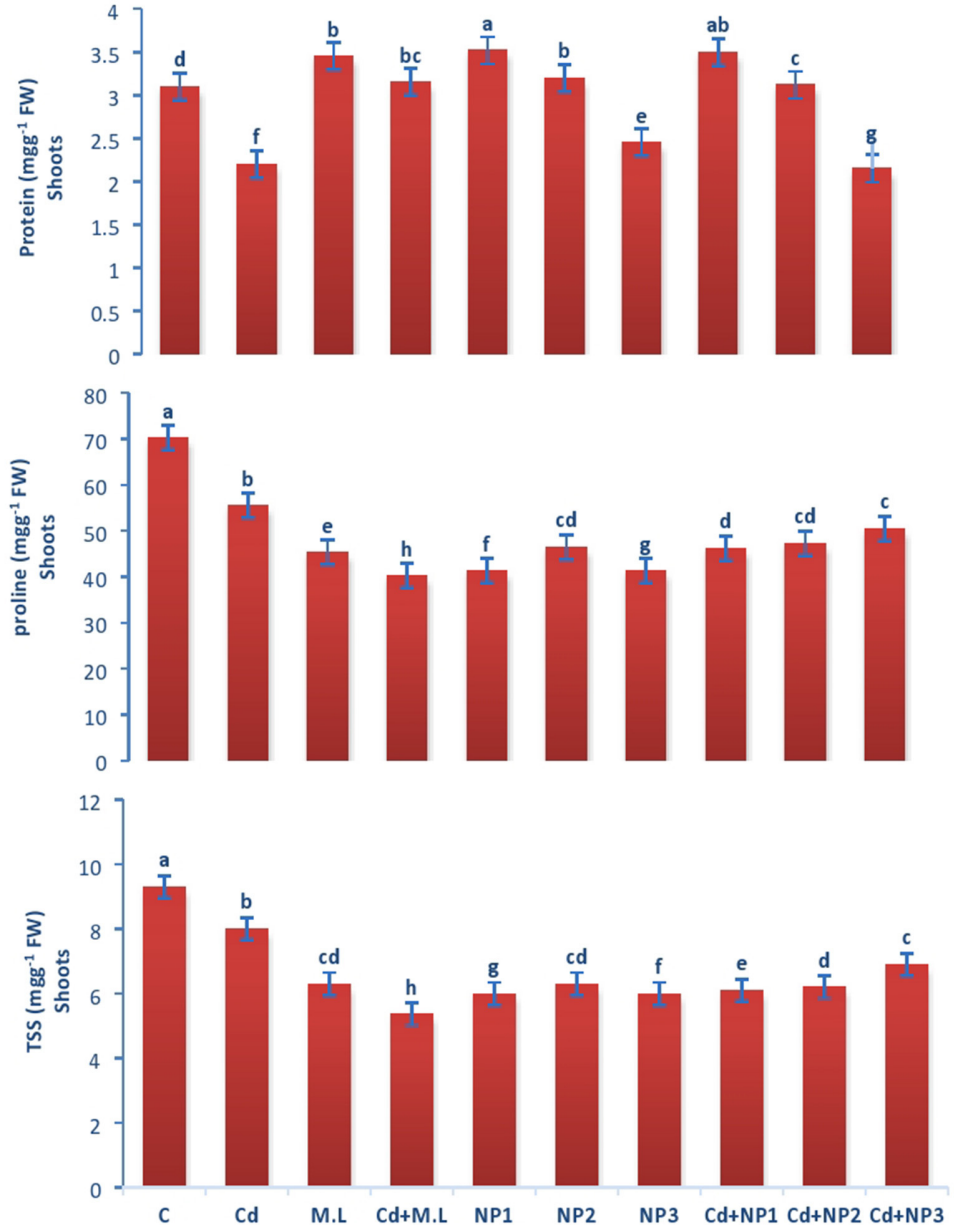
Effects of MZnO–NPs on protein, proline, and TSS under Cd stress in Linseed shoots. Different letters indicate significant difference between the treatments. Data are means ± SE (*n* = 5). Non-identical letters specify significant difference at *P* ≤ 0.05. C, Control; Cd, 100 mg kg^−1^ Cd; M. L, *Moringa oleifera* leaves; NP1, 100 mg L^−1^ MZnO-NPs; NP2, 500 mg L^−1^ MZnO-NPs; NP3, 1 g L^−1^ MZnO-NPs.

The amount of proline in roots enhanced in control plants in contrast to Cd-stressed plants as shown in Figures 3, 4. However, the levels of proline decreased when plants were supplemented with 2.8 g/100 ml *M. oleifera* leaf extract. The amount of proline is not significantly different among all the MZnO NPs levels. However, enhancement in the level of proline was observed in NP3 (1 g/L) MZnO NPs under Cd stress compared with that in the plants supplemented with MZnO NPs alone.

The amount of proline in shoots increased in control plants in contrast to Cd-stressed plants as shown in Figure 4. However, the levels of proline decreased when plants were supplemented with 2.8 g/100 ml *M. oleifera* leaf extract. The amount of proline is not significantly different among all the MZnO NPs levels. However, enhancement in the level of proline was observed in NP1 (100 mg/L) MZnO NPs under Cd stress compared with that in the plants supplemented with MZnO NPs alone.

### Evaluation of Catalase Enzyme Activity in Linseed Roots and Shoots Under Cadmium Stress

**Figure 5:**
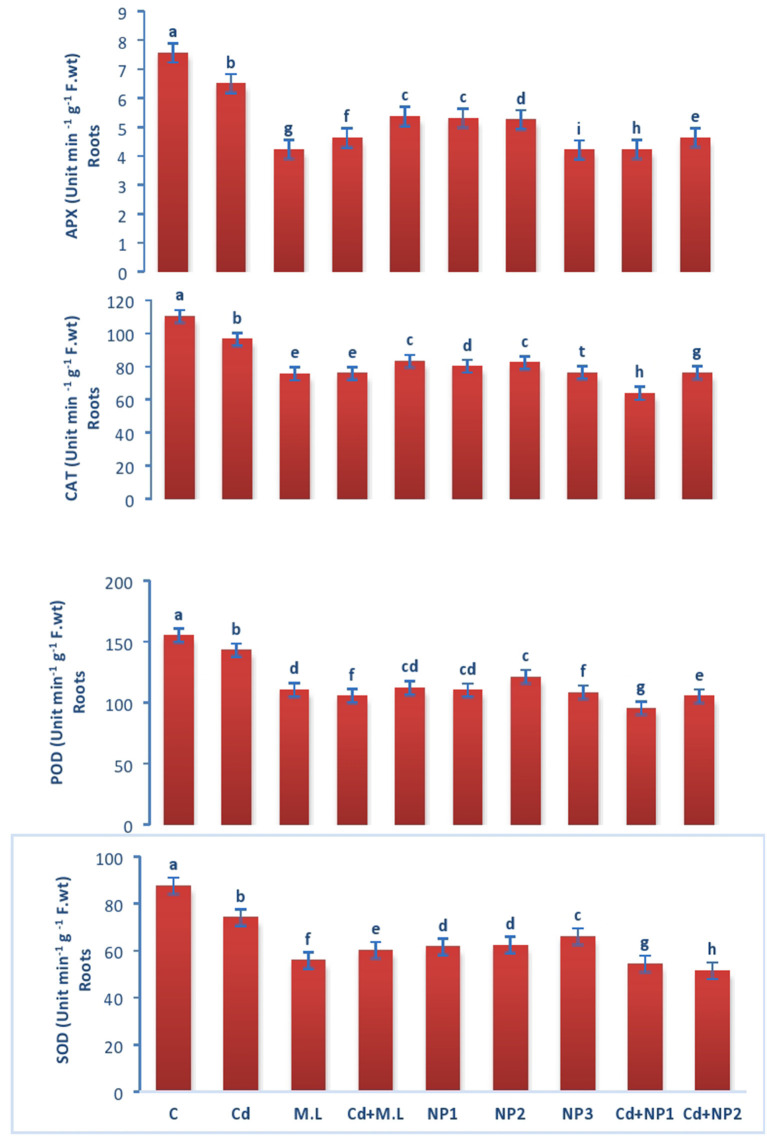
Effects of MZnO–NPs on APX, CAT, POD, and SOD under Cd stress in Linseed roots. Different letters indicate significant difference between the treatments. Data are means ± SE (*n* = 5). Non-identical letters specify significant difference at *P* ≤ 0.05. C, Control, Cd, 100 mg kg^−1^ Cd, M. L, *Moringa oleifera* leaves; NP1, 100 mg L^−1^ MZnO-NPs; NP2, 500 mg L^−1^ MZnO-NPs; NP3, 1 g L^−1^ MZnO-NPs.

**Figure 6:**
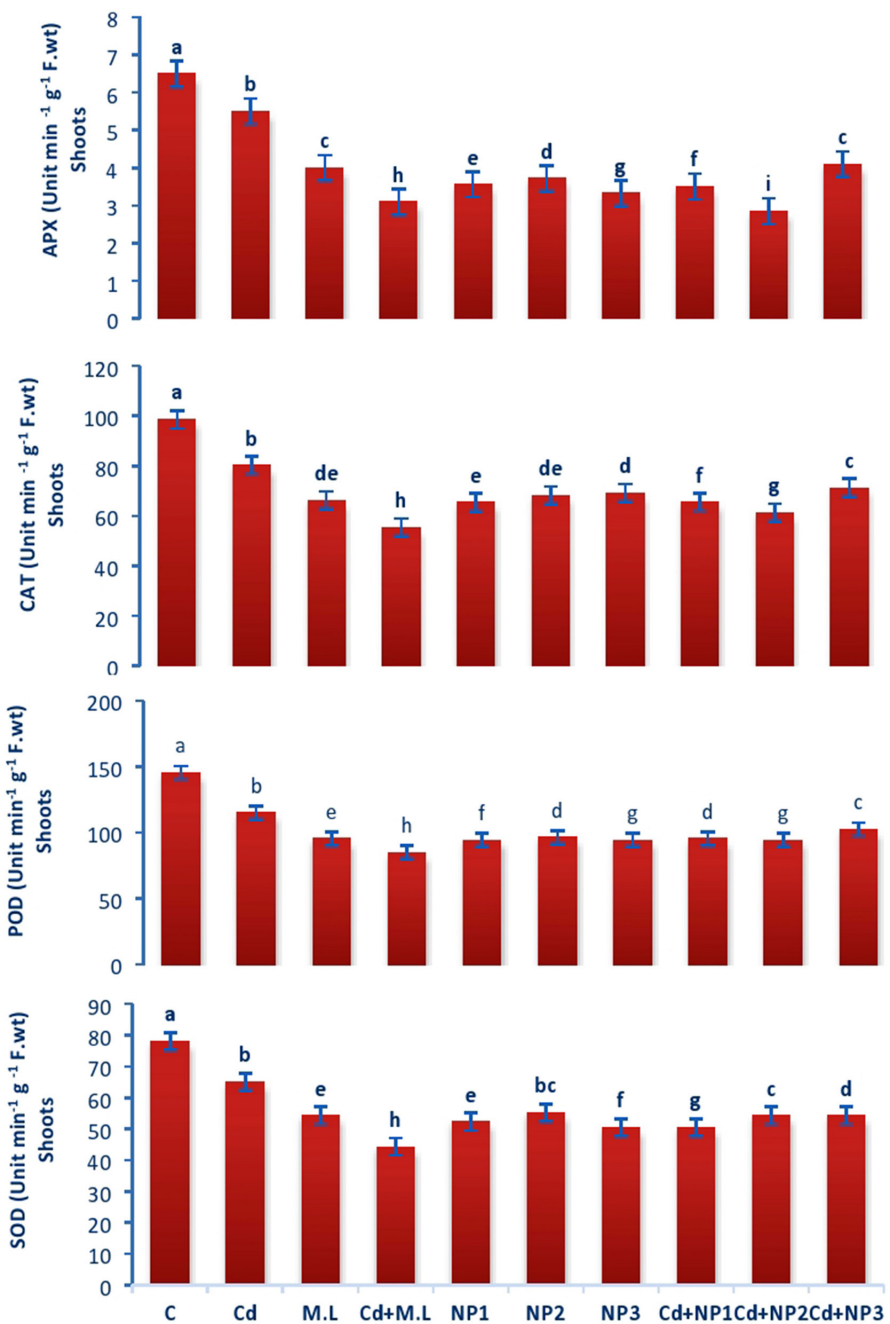
Effects of MZnO–NPs on APX, CAT, POD, and SOD under Cd stress in Linseed shoots. Different letters indicate significant difference between the treatments. Data are means ± SE (*n* = 5). Non-identical letters specify significant difference at *P* ≤ 0.05. C, Control; Cd, 100 mg kg^−1^ Cd; M. L, *Moringa oleifera* leaves; NP1, 100 mg L^−1^ MZnO-NPs; NP2, 500 mg L^−1^ MZnO-NPs; NP3, 1 g L^−1^ MZnO-NPs.

The activity of CAT enzyme in untreated plants was found to be increased compared with Cd-treated plants roots (Figure 5). However, levels of CAT were declined when they were supplemented with alone *M. oleifera* leaf extract, relative to untreated and Cd-stressed plants alone. While in case of NPS application alone, no significant difference was observed among the all levels of MZnO NPs. The activity of CAT enzyme in shoot tissues of untreated plants was increased relative to Cd-treated seedlings as shown in Figure 6. The application of *M. oleifera* leaf extract alone exhibits declined value of CAT enzyme activity compared with controlled and Cd-stressed seedlings, while in case of NPs application alone, no significant difference was observed among all the levels of MZnO NPs.

### Evaluation of Superoxide Dismutase Activity in Linseed Root and Shoots Under Cadmium Stress

Superoxide dismutase performance in untreated plants was found to be increased compared with Cd-treated plants roots (Figure 5). However, the levels of SOD were declined when they were supplemented with *M. oleifera* leaf extract alone, relative to untreated and Cd-stressed plants. While in case of NPs application alone, no significant difference was observed among the all levels of MZnO NPs. SOD performance in shoot tissues of untreated plants was increased relative Cd-treated plants seedlings in case of shoots as shown in Figure 6. The application of *M. oleifera* leaf extract alone exhibits declined value of SOD compared with controlled and Cd-stressed seedlings, while in case of NPs application alone, no significant difference was observed among the all levels of MZnO NPs.

### Evaluation of Peroxidase Dismutase Activity in Linseed Root and Shoots Under Cadmium Stress

The activity of POD in untreated plants was found to be increased compared with Cd-treated plants roots (Figure 5). However, levels of POD were declined when they were supplemented with *M. oleifera* leaf extract alone, relative to untreated and Cd-stressed plants. While in case of NPs application alone, no significant difference was observed among the all levels of MZnO NPs. The activity of POD in shoot tissues of untreated plants was increased relative to Cd-treated seedlings (Figure 6). The application of *M. oleifera* leaf extract alone exhibits declined value of POD compared with controlled and Cd-stressed seedlings, while in case of NPs application alone, no significant difference was observed among the all levels of MZnO NPs.

### Evaluation of Ascorbate Peroxidase Activity in Linseed Shoots Under Cadmium Stress

The activity of APX in untreated plants was found to be increased compared with Cd-treated plants roots (Figure 5). However, the levels of APX were declined when they were supplemented with alone M.L extract, relative to untreated and Cd-stressed plants. While in case of NPs application alone, no significant difference was observed among the all levels of MZnO NPs. The activity of ascorbate peroxidase (APX) in shoot tissues of untreated plants was increased relative to Cd-treated seedlings (Figure 6). The application of *M. oleifera* leaf extract alone exhibits declined value of APX compared with controlled seedlings. While in case of NPs application alone, no significant difference was observed among the all levels of MZnO NPs. APX activity increased under all Cd levels when supplemented with NP2 (500 mg/L) and NP3 (1 g/L).

### Evaluation of Electrolyte Leakage in Linseed Root and Shoots Under Cadmium Stress

**Figure 7:**
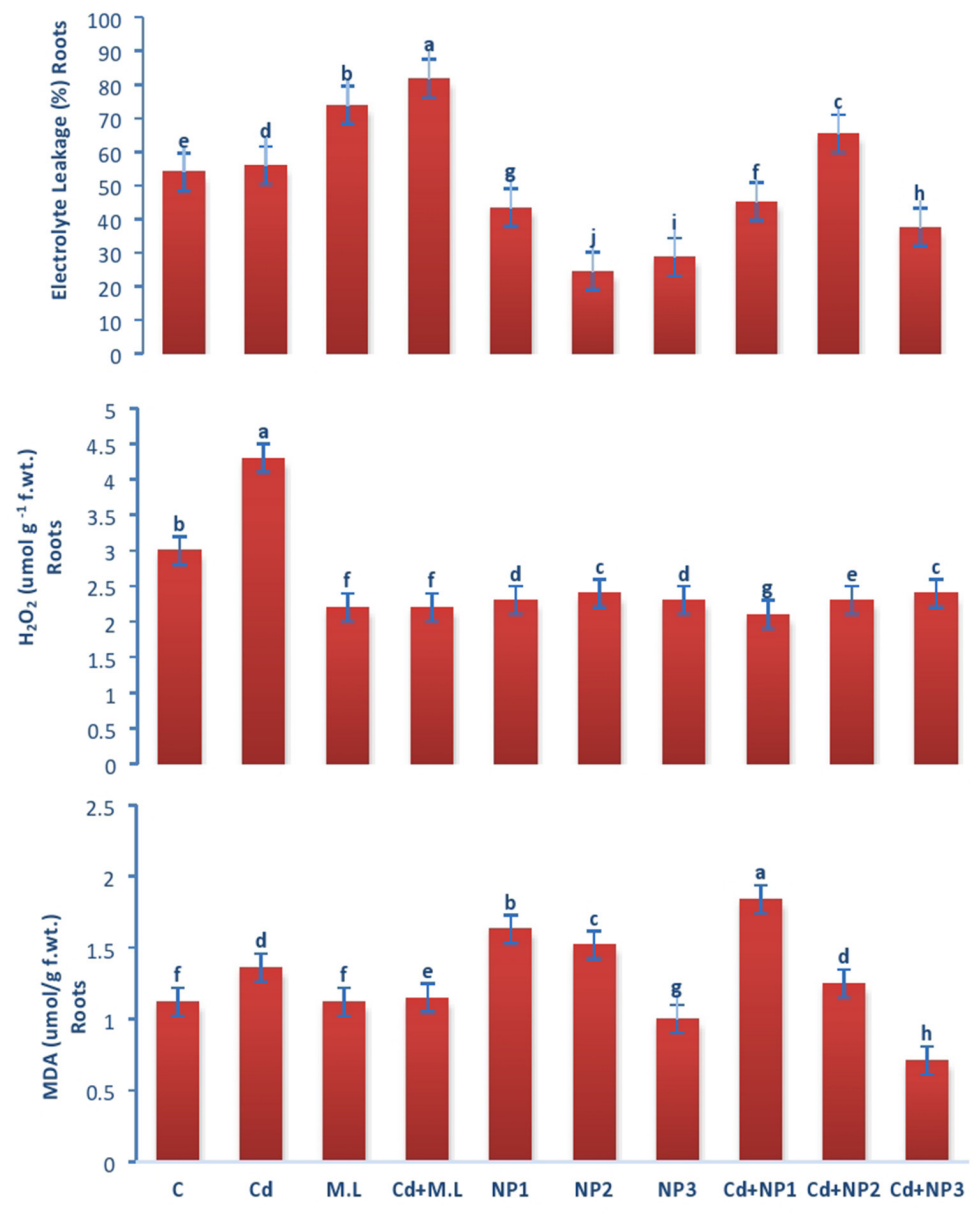
Effects of MZnO–NPs on EL, H_2_O_2_, and MDA under Cd stress in Linseed roots. Different letters indicate significant difference between the treatments. Data are means ± SE (*n* = 5). Non-identical letters specify significant difference at *P* ≤ 0.05. C, Control; Cd, 100 mg/kg Cd; M. L, *Moringa oleifera* leaves; NP1, 100 mg/L MZnO-NPs; NP2, 500 mg/L MZnO-NPs; NP3, 1 g/L MZnO-NPs.

The electrolyte leakage declined in nontreated plants compared with Cd-treated plants as shown in Figure 7. Exogenous application of NPs alone without Cd stress increased the EL at NP1 (100 mg/L) level while decreased the EL at NP3 (1 g/L) level. Under Cd stress, exogenous application of MZnO NPs deceased the EL relative to those plants treated with Cd alone.

### Evaluation of Malondialdehyde Acetate Content in Linseed Root and Shoots Under Cadmium Stress

**Figure 8:**
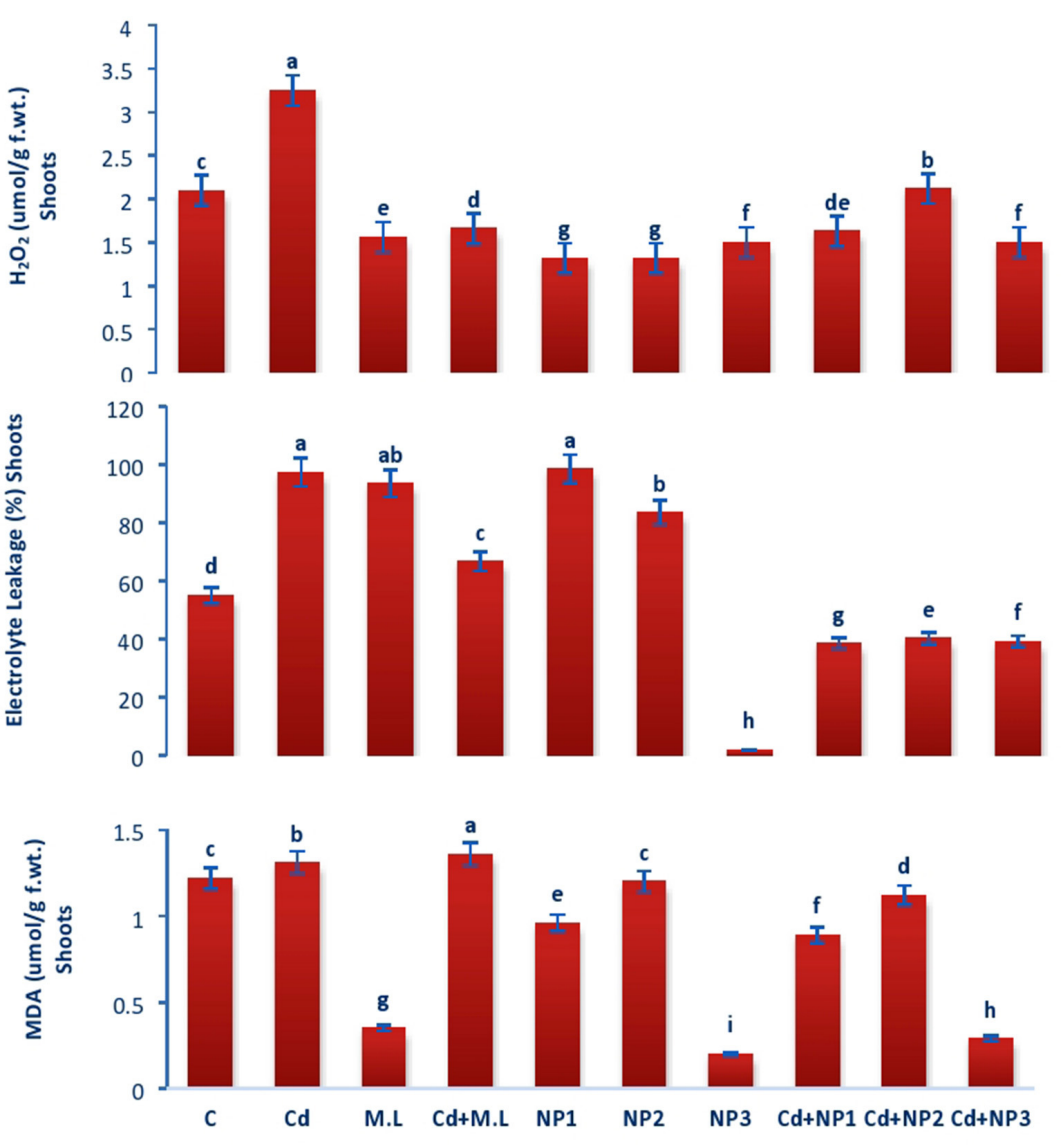
Effects of MZnO–NPs on EL, H_2_O_2_, and MDA under Cd stress in Linseed shoots. Different letters indicate significant difference between the treatments. Data are means ± SE (*n* = 5). Non-identical letters specify significant difference at *P* ≤ 0.05. C, Control; Cd, 100 mg kg^−1^ Cd; M. L, *Moringa oleifera* leaves; NP1, 100 mg L^−1^ MZnO-NPs; NP2, 500 mg L^−1^ MZnO-NPs; NP3, 1 g L^−1^ MZnO-NPs.

Malondialdehyde content in shoots increased with Cd over the control as shown in Figure 8. The application of *M. oleifera* leaf extract (2.8 g/100 ml) shows accumulation of MDA content with the treatment of Cd levels over the alone *M. oleifera* leaf extract supplemented root and shoot. However, exogenous application of NP2 (500 mg/L) enhanced MDA content only in non-Cd-treated plants as compared with those of plants subjected to NP1 (100 mg/L) and NP3 (1 g /L) alone.

### Evaluation of Hydrogen Peroxide in Linseed Root and Shoots Under Cadmium Stress

In case of roots, H_2_O_2_ content increased with Cd levels over the control as shown in Figure 7. The application of *M. oleifera* leaf extract (2.8 g/100 ml) shows accumulation of H_2_O_2_ content with the treatment of Cd (Figure 7). The application of *M. oleifera* leaf extract (2.8 g/100 ml) shows accumulation of H_2_O_2_ content with the treatment of Cd levels over the *M. oleifera* leaf extract-supplemented plants alone. However, exogenous application of NP3 (1 g/L) enhanced H_2_O_2_ content only in nonstressed plants as compared with those of plants subjected to NP1 (100 mg/L) and NP2 (500 mg/L) alone.

## Discussion

Heavy metals and metalloids are extremely poisonous to all species, including humans, causing major problems with normal metabolism and cellular functioning. Cd is continuously introduced to the soil from many sources, and because it is an analog of phosphate fertilizer, it is easily translocated by plants (di Toppi and Gabbrielli, 1999; Rao et al., 2010; Dixit et al., 2011). Excessive usage of Cd-rich sewage sludge and fertilizer has had a negative influence on human health; it accumulates extensively in edible plant parts (Karthik et al., 2017). The recent study investigated to examine the potentiality of exogenous MZnO NPs application in alleviation of Cd uptake and growth in linseed. In this study, Cd declined growth significantly, but exogenously applied MZnO NPs was effective in mitigating the Cd-induced negative effects and improving growth under normal, as well as Cd-induced stress circumstances. Similar to our findings, Paparella et al. (2015) and Rizwan et al. (2018) found that ZnO NPs and Fe NPs promoted wheat growth and development.

Another possible cause for higher plant growth with nanomaterials is that nutrients like Zn encourage plant chlorophyll production (Briat et al., 2007). The key component of chloroplast, i.e., chlorophyll, is positively associated to the photosynthetic rate of plants. Cd and many other metals have a considerable effect on leaf chlorophyll concentration, which is the principal signs of metal toxicity in plants (Rizwan et al., 2016). Variation in chlorophyll concentration represents plants growth as well as plant reaction to environmental change. Cd revealed in the results, there was enhancement in chlorophyll content and photosynthetic parameters in linseed leaves by Moringa-stabilized ZnO NPs (1 g/L) as shown in Table 2. Rizwan et al. (2018) revealed that zinc oxide and Fe NPs both boost chlorophyll content in Cd-stressed plants.

Same findings were found by Mahakham et al. (2017), who discovered that a rise in chlorophyll concentrations in NPs-primed plants can be associated to increased water and nutrient absorption, resulting in increased physiological activity of the plants. Some previous studies have revealed the efficacy of NPs in improving photosynthesis in plants, with dose-dependent and plant species-dependent responses (Sreelatha et al., 2011; Rizwan et al., 2017b). In our research, EL was decreased in roots and shoots tissues after supplementation of MZnO NPs, whereas leaf water content was also increased in these seedlings. This could be attributed to increased water and mineral element absorption in the presence of ZnO NPs (Pervaiz et al., 2020).

Cadmium stress decreases the content of essential osmolytes, particularly proline, total soluble sugar, and protein in this study. However, MZnO NPs supplemented Cd-stressed seedlings increased these osmolytes. Similar findings were found to be in agreement with Ahanger and Agarwal (2017a,b), who indicated that these osmolytes are significant in mitigating stress-induced damage. As a result, an enhancement in their content under hazardous state is a natural defensive mechanism in plants. According to Choudhury et al. (2011), Cd stress increases proline accumulation in soybean. Siddiqui et al. (2015) found that Cd stress increased proline accumulation in *Withania somnifera* and *Oryza sativa*. They stimulate proline buildup, and GB preserves biochemical processes, enhances ROS scavenging, and regulates redox homeostasis and the activities of several enzymes (Kaya et al., 2020).

The growing severity of As stress in soybean plants resulted in elevated amounts of H_2_O_2_, MDA, and EL. Other plants cultivated under other conditions have likewise shown increased H_2_O_2_ and MDA acetate buildup, as well as enhanced EL (Ahanger and Agarwal, 2017a,b; Ahmad et al., 2018; Kaya et al., 2020). Supplementation of MZnO NPs (1 g/L) in our research lowered the accumulation of MDA and H_2_O_2_ contents, and our results correlate with the findings of some earlier reports with different plant species (Hussain et al., 2016; Rizwan et al., 2017a,b, 2019a,b; Venkatachalam et al., 2017; Pervaiz et al., 2020).

Plants have an antioxidant system containing enzymatic and nonenzymatic antioxidants, the latter of which include ascorbic acid (ASA), glutathione (GSH), alpha-tocopherols, and phenolic chemicals (Ahanger and Agarwal, 2017a). Cd stress increased the activity of antioxidant enzymes, which is consistent with the results of Talukdar (2013), Yadav and Srivastava (2015), and Siddiqui et al. (2015). Under Cd stress, Lu et al. (2019) found increased activity of SOD, CAT, POD and APX in tartary buckwheat. The level of NPs increased after MznO NPs administration and antioxidant enzyme activation.

Various studies reported similar findings; López-Moreno et al. (2010) and Ghosh et al. (2016) both noticed an enhancement in antioxidant defense system under ZnO NPs exposure, which is a result of gene expression (Nair and Chung, 2014). Many other plant species react similarly to ZnO NPs by increasing the effectiveness of antioxidant systems and the functions of peroxidase, CAT enzyme, and superoxide dismutase (Singh et al., 2013; Soliman et al., 2015; Abdel Latef et al., 2017; Rizwan et al., 2017a,b, 2019a,b; Kantabathini et al., 2018; Wang et al., 2018). Many authors have found elevated SOD, CAT, APX, and GR activities produced through ZnO–NP in various plants, including Tripathi et al. (2015) in pea, Hernandez-Viezcas et al. (2011) in *Prosopis juliflora*, and Krishnaraj et al. (2012) in *Bacopa monnieri*. These increased antioxidant functions aided the plants in scavenging the additional ROS produced in response to stressful signals.

## Conclusion

Cadmium stress reduced growth and physiochemical properties of linseed plants. However, application of MZnO NPs reduced Cd accumulation in linseed. The MZnO NPs raised Zn concentrations, indicating that NPs might be employed in the bio-fortification of cereals while lowering Cd levels. As a result, this technology could be a greener alternative to traditional methods used to lower Cd levels in plants and, eventually, in human beings. Synergistic application of *M. oleifera* leaf extract and ZnO NP protects photosynthesis and ameliorates the deleterious effects on the osmolyte component by restricting the accumulation of Cd thereby minimizing the chance of oxidative stress. Furthermore, investigations in real-world field circumstances and with different plants are required to validate these findings. Synergistic application of *M. oleifera* leaf extract and ZnO NP increased the activity of antioxidant enzymes, providing additional strength to reduce ROS generation.

## Data Availability Statement

The raw data supporting the conclusions of this article will be made available by the authors, without undue reservation.

## Author Contributions

FA: experimentation. MR: conceptualization. RS: formatting and software. AAS: review and formatting, statistical analysis. ANS: drafting. GN: formatting and review. AM: formatting. AT: statistical analysis and funding acquisition. HK: funding acquisition and writing-review and editing. ED: formal analysis and funding acquisition. HE: statistical analysis and funding acquisition. All authors contributed to the article and approved the submitted version.

## Funding

This study was funded by Taif University Researchers Supporting Project number (TURSP-2020/85), Taif University, Taif, Saudi Arabia.

## Conflict of Interest

The authors declare that the research was conducted in the absence of any commercial or financial relationships that could be construed as a potential conflict of interest.

## Publisher's Note

All claims expressed in this article are solely those of the authors and do not necessarily represent those of their affiliated organizations, or those of the publisher, the editors and the reviewers. Any product that may be evaluated in this article, or claim that may be made by its manufacturer, is not guaranteed or endorsed by the publisher.
